# Clinical Course and Outcome of Cardiovascular Manifestations in Children With Multisystem Inflammatory Syndrome Associated With SARS-CoV-2 Infection in Georgia

**DOI:** 10.7759/cureus.38555

**Published:** 2023-05-04

**Authors:** Sofia Phirtskhalava, Elene Shavgulidze, Aadil Ashraf Ahmed Shaikh, Farah Marikar, Ketevan Kalatozishvili, Ana Maghradze, Ivane Chkhaidze

**Affiliations:** 1 American MD Program, Tbilisi State Medical University, Tbilisi, GEO; 2 Faculty of Psychology and Educational Sciences, Tbilisi State University, Tbilisi, GEO; 3 Pediatrics, M.Iashvili Children’s Central Hospital, Tbilisi, GEO

**Keywords:** cardiology, arrhythmias, valvular insufficiency, coronary dilation, mis-c, sars-cov-2

## Abstract

A SARS-CoV-2 infection is usually characterized by a very mild clinical course in the pediatric population. However, children can be severely affected, and clinical manifestations may differ from adults, mainly in terms of post-COVID-19 infection complications already known as multisystem inflammatory syndrome in children (MIS-C). As the name suggests, this condition involves many systems, including the cardiovascular system, clinical manifestations of which include myocarditis, coronary artery aneurysms, conduction abnormalities, and arrhythmias.

This research aims to define the cardiac manifestations caused by multi-inflammatory processes occurring after acute SARS-CoV-2 infection, possibly find a correlation between a certain cardiac abnormality and inflammatory markers, and evaluate the dynamics of cardiovascular complications and how treatment affects it.

From February 2020 to March 2022, 103 patients with MIS-C were hospitalized and treated at M.Iashvili Children’s Central Hospital, Tbilisi, Georgia. Based on our results, 55% of them had cardiovascular involvement with various manifestations involving coronary artery dilation, valvular insufficiencies, heart rate abnormalities, and pericardial effusion. Our study revealed that only one statistically significant correlation was observed between D-dimer levels and heart rate abnormalities, but there was no correlation between these two values.

All of the MIS-C patients reported in our study have received standardized treatment courses with steroids, intravenous immune globulin (IVIG), or IVIG combined with steroids; each patient’s illness has resolved without any sequelae, and cardiac manifestations have returned to baseline. Nevertheless, systematic longer-term follow-up is needed to provide clarity on the evolution of medium- and long-term cardiac outcomes in MIS-C.

## Introduction

The first cases of COVID-19 were reported in Europe on January 24, 2020 [1]. The initial experience and expectation were that children would be minimally affected in comparison with the widespread morbidity and mortality seen in adults [2]. However, toward the end of April 2020, the unexpected emergence of a novel inflammatory shock syndrome in children, which shares clinical features with other well-known syndromes including Kawasaki disease (KD), was documented [3]. The Centers for Disease Control and Prevention (CDC) termed this condition as multisystem inflammatory syndrome in children (MIS-C) associated with COVID-19 infection and provided a detailed case definition [4]. According to it, multiorgan (cardiac, gastrointestinal, mucocutaneous, and hematologic) involvement is a cardinal sign of the described syndrome. To this day, the pathophysiology of MIS-C is not completely understood, but it is considered to be a hyperimmune state resulting from cytokine storm and circulating immune complexes [5]. The exact mechanism by which COVID-19 triggers an abnormal immune response is unknown and is the subject of active investigation. On the other hand, thorough investigations in search of uncovering the underlying mechanisms of MIS-C have led researchers to investigate the clinical manifestations of MIS-C, which revealed that MIS-C is known to affect the cardiovascular system in up to 68-70% of cases [6]. Cardiac dysfunction may result from a multitude of causes, some of which include ongoing systemic inflammation, stress-induced cardiomyopathy, hypoxic injury, coronary artery abnormalities, or a mixture of the abovementioned [7]. Cardiac involvement in MIS-C can range from myocardial dysfunction and arrhythmias to more severe conditions, such as cardiogenic shock [8]. For example, according to one study, coronary artery aneurysms were documented at 4% [9], while the other study showed the prevalence to be 8% [10]. Reduced left ventricular (LV) ejection fraction (EF) was also observed and was present in more than 50% of patients; troponin levels were raised in the majority of them [3]. The mechanism for the change in ventricular function appears to be multifactorial, including the events of cardiomyocyte injury and generalized inflammation [11]. Other studies have described 7-60% of patients having rhythm abnormalities of variable severity [7] and 21% of patients having pericardial effusions [3].

From February 2020 to March 2022, 103 patients with MIS-C were hospitalized and treated at M.Iashvili Children’s Central Hospital, Tbilisi, Georgia. The main aim of our study is oriented toward documenting and following up on cardiovascular manifestations in these patients according to all the data obtained during the hospitalization and from further one-year follow-up sessions.

## Materials and methods

The given research represents a retrospective cohort study with prospective elements because we followed the patients in real time as well as reviewed their medical record data retrospectively. The latter was obtained from the database of 103 patients diagnosed with MIS-C (according to the criteria presented by CDC) hospitalized at M.Iashvili Children’s Central Hospital.

The hospital database has been created according to the following: patients’ names/surnames (which were removed before starting the data analysis), demographic data (age and gender), symptoms on admission (e.g., cough, dyspnea, and palpitations), time period from past COVID-19 infection, degree of past COVID-19 infection (asymptomatic and symptomatic), presence or absence of positive PCR/serology/antigen test, dates of admission and discharge, clinical findings during hospitalization (degree of fever, complete blood count (CBC), liver function tests (LFT), C-reactive protein (CRP), erythrocyte sedimentation rate (ESR), fibrinogen, D-dimer, ferritin, procalcitonin, lactate dehydrogenase (LDH), albumin, brain natriuretic peptide (BNP), interleukin 6 (IL-6), and findings of electrocardiogram, echocardiography, and chest X-ray), doctor providing treatment, treatment provided (IVIG + steroid, only steroid), and duration of treatment.

The study population consists of Georgian pediatric population of one to 17 years old diagnosed with MIS-C during the period of February 2020-March 2022. The most attention is drawn to children diagnosed with MIS-C and documented cardiovascular complications, under which the following conditions are meant: coronary artery dilation and aneurysms, conduction abnormalities, arrhythmias, pericardial effusions, and valvular diseases.

Patients with major preexisting comorbidities, present congenital heart disease, and/or preexisting cardiovascular disease have been excluded from the study, as the goal is to document cardiovascular findings exclusively related to SARS-CoV-2-associated MIS-C.

Results of the abovementioned clinical investigations have been re-checked during follow-up sessions within one year from discharge for evaluation of treatment efficacy. The follow-up monitoring for the patients with cardiovascular complications was planned according to the schedule of the national protocol created based on the CDC guidelines. For follow-up sessions, patients’ parents have been contacted and asked to come to the hospital for consultation and the required workup. Echocardiography was performed two weeks after discharge for patients who did not have coronary artery dilation during the hospitalization. For those who had any degree of coronary artery dilation, they would undergo echocardiography multiple times: once every two to three days until the normal findings were observed, followed by monitoring every two weeks for six weeks in total. All the other patients would undergo follow-up via phone call three months after discharge, unless visiting the hospital was required.

In our subjects, not all the abovementioned data results were available simultaneously; therefore, based on the given database, we decided to emphasize only CRP, D-dimer, and ferritin levels as the markers representing inflammation. Our goal was to document and find a correlation, if any, between these inflammatory markers and the already mentioned cardiovascular findings. For this purpose, statistical analysis of the paper, specifically correlation analysis, was performed using Statistical Package for the Social Sciences (SPSS) software, which enabled us to calculate the Pearson correlation coefficient and derive p values for the research data. Tables describing the correlation analysis were developed using the SPSS platform as well. Figures showcased in the paper were constructed using the Google Sheet platform.

It is worth mentioning that the study does not include the names, last names, or initials of the study subjects. Therefore, as there is no information in this study that would facilitate revealing their identities, no consent was taken from patients or their caregivers. The study received ethical clearance from the institutional review board.

## Results

We identified 103 consecutive MIS-C patients. The median age at admission was seven years, ranging from one to 17 years (interquartile range (IQR), nine). Cardiovascular involvement was observed in 55% of patients in our cohort. A high fever ranging from 39°C to 41°C was present in all patients (100%).

The abovementioned 57 patients (55%) were observed to have cardiac manifestations of MIS-C that included mitral and tricuspid valve insufficiencies, coronary artery dilation, nonspecific ST wave changes, arrhythmias, conduction abnormalities, heart block in three patients (0.09%), reduced LVEF (1%), or even pericardial effusion (14%).

**Figure 1:**
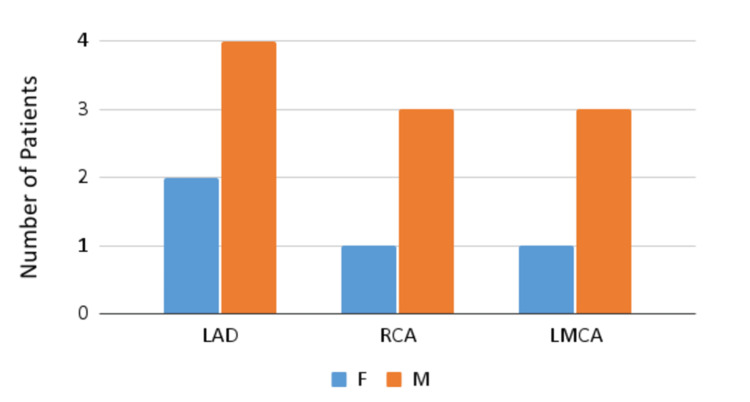
Coronary artery dilation LAD, left anterior descending artery; RCA, right coronary artery; LMCA, left main coronary artery; F, female; M, male

Echocardiography revealed coronary artery dilation in a total of eight (14.8%) patients. Six patients out of eight developed dilation of the left anterior descending artery (LAD; mean z-score of 3.42), four out of eight developed left main coronary artery (LMCA) dilation (mean z-score of 2.8225), and right coronary artery (RCA) dilation (mean z-score of 2.8375) was observed in four out of eight patients. The abovementioned patients were also presented with a combination of these artery dilation (Figure 1).

**Figure 2:**
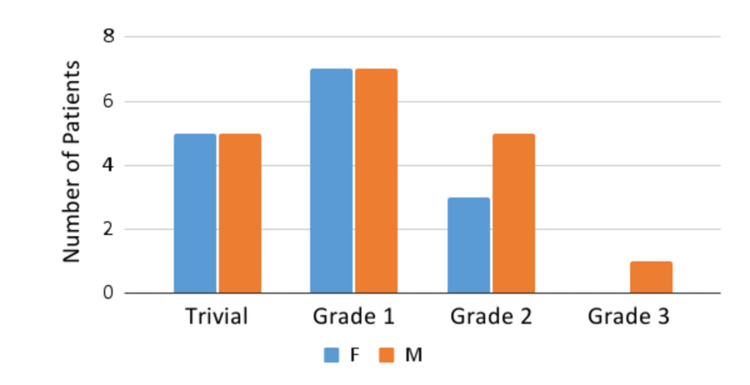
Mitral valve insufficiency F, female; M, male

**Figure 3:**
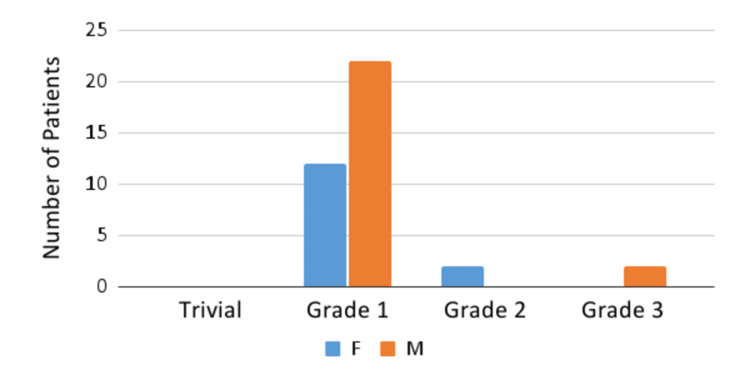
Tricuspid valve insufficiency F, female; M, male

Valvular insufficiencies were observed in a total of 44 (77%) patients. Some of the patients in the cohort have demonstrated combined mitral and tricuspid valvular regurgitations. A total of 33 patients out of 57 (61.1%) have developed mitral valve insufficiency, the severity of which was defined based on Doppler color flow mapping (trivial, grade one (mild), grade two (moderate), and grade three (moderate-to-severe)). Tricuspid valve insufficiency was observed in a total of 38 patients (70.3%), the severity of which was again defined based on Doppler color flow mapping (trivial, grade one (mild), grade two (moderate), and grade three (moderate-to-severe)) (Figures 2, 3).

**Figure 4:**
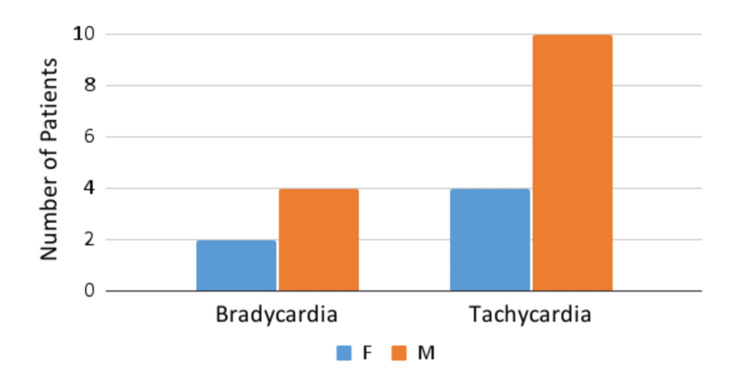
Heart rate abnormalities F, female; M, male

In addition to the abovementioned findings, heart rate abnormalities were also described in the cohort. A total of 20 (37%) patients have demonstrated cardiac pulse derangements, including bradycardia (pulse range, 50-94; mean age, four years) and tachycardia (pulse range, 104-175; mean age, 9.2 years). As heart rate differs according to the age of the patient, we have used a special chart [12] to define the normal ranges of heart rate, excluding tachycardic findings caused by high temperature or crying (Figure 4).

As anticipated, laboratory testing showed an increase of cardiac biomarkers, e.g., troponin, as well as the elevation of inflammatory markers (ferritin, CRP, ESR, and D-dimer) and neutrophilia. One of the main objectives of this research was to identify possible correlations between specific cardiovascular manifestations and inflammatory markers. First, the correlation between D-dimer levels and mitral insufficiency was defined. The Pearson correlation coefficient was calculated to be 0.251, and the p value was 0.166 (Table 1).

**Table 1:** Correlation between D-dimer levels and mitral valve insufficiency grade Sig. (two-tailed), p value; N, number of patients

		D-dimer levels	Mitral valve insufficiency grade
D-dimer levels	Pearson correlation	1	0.251
	Sig. (two-tailed)		0.166
	N	48	32
Mitral valve insufficiency grade	Pearson correlation	0.251	1
	Sig. (two-tailed)	0.166	
	N	32	33

The same manipulation was performed for D-dimer levels and tricuspid insufficiency, yielding a Pearson correlation coefficient of -0.087 and a p value of 0.620 (Table 2).

**Table 2:** Correlation between D-dimer levels and tricuspid valve insufficiency grade Sig. (two-tailed), p value; N, number of patients

		D-dimer levels	Tricuspid valve insufficiency grade
D-dimer levels	Pearson correlation	1	-0.087
	Sig. (two-tailed)		0.620
	N	48	35
Tricuspid valve insufficiency grade	Pearson correlation	-0.087	1
	Sig. (two-tailed)	0.620	
	N	35	36

Next, we correlated D-dimer levels with troponin levels in the cohort, which yielded a correlation coefficient of -0.214 and a p value of 0.365 (Table 3).

**Table 3:** Correlation between D-dimer levels and troponin levels Sig. (two-tailed), p value; N, number of patients

		D-dimer levels	Troponin levels
D-dimer levels	Pearson correlation	1	-0.214
	Sig. (two-tailed)		0.365
	N	48	20
Troponin levels	Pearson correlation	-0.214	1
	Sig. (two-tailed)	0.365	
	N	20	20

Similarly, the correlation between D-dimer levels and heart rate was showcased, resulting in a correlation coefficient of 0.390 and a p value of 0.027 (Table 4). 

**Table 4:** Correlation between D-dimer levels and heart rate Sig. (two-tailed), p value; N, number of patients *Correlation is significant at the 0.05 level (two-tailed)

		D-dimer levels	Heart rate
D-dimer levels	Pearson correlation	1	0.390*
	Sig. (two-tailed)		0.027
	N	48	32
Heart rate	Pearson correlation	0.390*	1
	Sig. (two-tailed)	0.027	
	N	32	34

**Figure 5:**
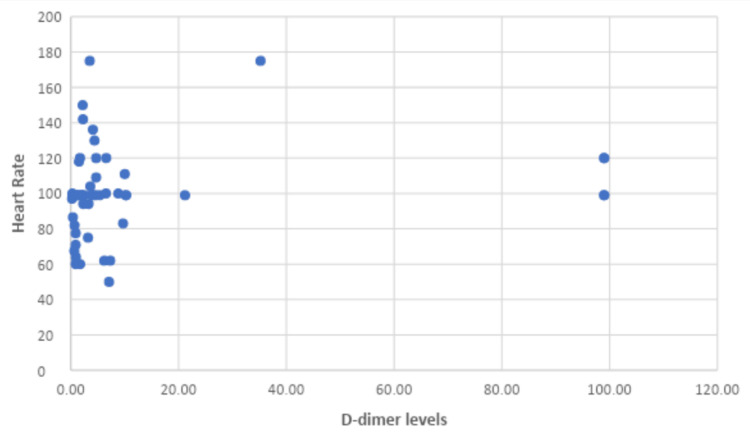
Correlation between D-dimer levels and heart rate

Therefore, a statistically significant correlation was observed between D-dimer levels and heart rate abnormalities, but the corresponding graph shows no correlation between these two values (Figure 5).

A correlation analysis was performed for CRP and cardiac manifestations. CRP levels were correlated with mitral valve insufficiency. Results showed a Pearson correlation coefficient of 0.083 and a p value of 0.669 (Table 5).

**Table 5:** Correlation between CRP levels and mitral valve insufficiency grade CRP, C-reactive protein; Sig. (two-tailed), p value; N, number of patients

		CRP levels	Mitral valve insufficiency grade
CRP levels	Pearson correlation	1	0.083
	Sig. (two-tailed)		0.669
	N	45	29
Mitral valve insufficiency grade	Pearson correlation	0.083	1
	Sig. (two-tailed)	0.669	
	N	29	33

Similar manipulation was performed for CRP levels and tricuspid insufficiency, which yielded a correlation coefficient of -0.118 and a p value of 0.519 (Table 6).

**Table 6:** Correlation between CRP levels and tricuspid valve insufficiency grade CRP, C-reactive protein; Sig. (two-tailed), p value; N, number of patients

		CRP levels	Tricuspid valve insufficiency grade
CRP levels	Pearson correlation	1	-0.118
	Sig. (two-tailed)		0.519
	N	45	32
Tricuspid valve insufficiency grade	Pearson correlation	-0.118	1
	Sig. (two-tailed)	0.519	
	N	32	36

Next, troponin levels were correlated with CRP levels, which again yielded no statistically significant results (Pearson correlation, -0.234; p = 0.382) (Table 7).

**Table 7:** Correlation between CRP levels and troponin levels CRP, C-reactive protein; Sig. (two-tailed), p value; N, number of patients

		CRP levels	Troponin levels
CRP levels	Pearson correlation	1	-0.234
	Sig. (two-tailed)		0.382
	N	45	16
Troponin levels	Pearson correlation	-0.234	1
	Sig. (two-tailed)	0.382	
	N	16	20

Considering the statistically significant correlation between D-dimer levels and heart rate, we wanted to see if there was also any correlation between CRP levels and heart rate, but it resulted in a Pearson correlation of 0.022 and a p value of 0.910; therefore, the results did not show any statistical significance (Table 8).

**Table 8:** Correlation between CRP levels and heart rate CRP, C-reactive protein; Sig. (two-tailed), p value; N, number of patients

		CRP levels	Heart rate
CRP levels	Pearson correlation	1	0.022
	Sig. (two-tailed)		0.910
	N	45	29
Heart rate	Pearson correlation	0.022	1
	Sig. (two-tailed)	0.910	
	N	29	34

The third inflammatory marker for which we performed a correlation analysis was ferritin. Ferritin levels were correlated with mitral insufficiency, resulting in a Pearson correlation of 0.146 and a p value of 0.540 (Table 9).

**Table 9:** Correlation between ferritin levels and mitral valve insufficiency grade Sig. (two-tailed), p value; N, number of patients

		Ferritin levels	Mitral valve insufficiency grade
Ferritin levels	Pearson correlation	1	0.146
	Sig. (two-tailed)		0.540
	N	29	20
Mitral valve insufficiency grade	Pearson correlation	0.146	1
	Sig. (two-tailed)	0.540	
	N	20	33

Similarly, ferritin levels were correlated with tricuspid insufficiency, yielding no statistically significant results (Pearson correlation, 0.322; p = 0.167) (Table 10).

**Table 10:** Correlation between ferritin levels and tricuspid valve insufficiency grade Sig. (two-tailed), p value; N, number of patients

		Ferritin levels	Tricuspid valve insufficiency grade
Ferritin levels	Pearson correlation	1	0.322
	Sig. (two-tailed)		0.167
	N	29	20
Tricuspid valve insufficiency grade	Pearson correlation	0.322	1
	Sig. (two-tailed)	0.167	
	N	20	36

Next, troponin levels were correlated with ferritin, and again, this resulted in no statistical significance (Pearson correlation, -0.021; p = 0.936) (Table 11).

**Table 11:** Correlation between ferritin levels and troponin levels Sig. (two-tailed), p value; N, number of patients

		Ferritin levels	Troponin levels
Ferritin levels	Pearson correlation	1	-0.021
	Sig. (two-tailed)		0.936
	N	29	17
Troponin levels	Pearson correlation	-0.021	1
	Sig. (two-tailed)	0.936	
	N	17	20

Lastly, ferritin levels were correlated with heart rate, demonstrating a Pearson correlation of 0.168 and a p value of 0.5 (Table 12). 

**Table 12:** Correlation between ferritin levels and heart rate Sig. (two-tailed), p value; N, number of patients

		Ferritin levels	Heart rate
Ferritin levels	Pearson correlation	1	0.168
	Sig. (two-tailed)		0.505
	N	29	18
Heart rate	Pearson correlation	0.168	1
	Sig. (two-tailed)	0.505	
	N	18	34

## Discussion

MIS-C is now a well-defined pediatric disease that first emerged during the COVID-19 pandemic. It is characterized by a hyperinflammatory condition with multisystem involvement [13]. Cardiovascular involvement is common and can range from mild ventricular dysfunction to severe refractory cardiogenic shock, vasodilatory shock, or significant arrhythmia [7]. Various global health organizations like the CDC and the World Health Organization (WHO) have established diagnostic criteria for MIS-C, which include persistent fever (T > 38°C), lymphopenia or organ dysfunction, elevation of inflammatory markers, and epidemiological or laboratory evidence of SARS-CoV-2 infection [4,14].

In our cohort, males and females were equally affected by the disease. The median age of these patients diagnosed with MIS-C was seven years (a range of one to 17 years of age), compared to the median age of eight to nine years reported by other studies [10,15].

We have previously described our experience with 103 patients diagnosed with MIS-C, in whom cardiac involvement could be as high as 55%, lining up with a similar study that proves that this finding could range from 50% to 87% [11].

A total of eight patients (14.8%) in our cohort developed dilation of the LAD (mean z-score of 3.42), RCA (mean z-score of 2.8375), LMCA (mean z-score of 2.8225), or combination of these three arteries. Belhadjer et al. also reported that dilation of the coronary arteries (z-score >2 adjusted for body temperature) was found in 17% of patients, including five patients with LMCA and one patient with RCA [16]. In another study that aimed at analyzing the echocardiographic manifestations of MIS-C, coronary artery dilation was recorded in 4% of patients with a z-score of 3.15 in the acute phase [9]. One of the similar studies [17] also showed evidence of moderate (or worse) valvular regurgitation in 26.8% (80.7% in our study; 46 patients), myocardial dysfunction in 46.3% (this finding was not present in our study), and pericardial effusions in 31.3% (14% in our study).

Elevation of inflammatory markers and evidence of hyperinflammation were widely reported in patients with MIS-C [18,19]. Overall, CRP and ESR are highly elevated. Similarly, in our cohort, the median CRP level was 145 mg/dL with a range of 12-393 mg/dL (normal value, 0-10 mg/dL), and the median ESR level was 42 mm/hr with a range of 5-72 mm/hr (normal value, 0-10 mm/hr). Myocardial dysfunction has been described in a large proportion of children with MIS-C. In the first MIS-C case series reported in the UK in mid-April 2020, cardiac dysfunction was observed in 75% of patients [18]. Belhadjer et al. reported a selected cohort of 35 MIS-C patients who developed depressed LV systolic dysfunction with reduced EF of 30% in 10 out of 35; in addition, LV hypokinesis was global in 31 out of 35 patients [16]. In our study, less than 1% had a borderline LVEF (56% in an 11-year-old child, 56% being the lower limit of the normal value). Another large-scale meta-analysis focusing on myocardial involvement in MIS-C revealed that BNP levels in severe MIS-C patients were higher than in non-severe COVID-19 patients; however, no significant difference was observed between severe MIS-C and severe COVID-19-induced increases in BNP. This finding provides the possibility of linking management strategies in patients with severe COVID-19 and MIS-C and provides clarity on the immune-pathologic basis of cardiac injury [20].

BNP levels were not measured in most of the patients involved in our study. However, as described above, we attempted to show a correlation between certain inflammatory markers (CRP, D-dimers, and ferritin) and the severity of the cardiovascular findings. Considering the abovementioned results, statistical significance was reached solely between D-dimer levels and heart rate, and the corresponding graph shows no correlation between these two values. Other findings were not statistically significant; therefore, we cannot say if there is any connection between the given data. The reason for the abovementioned results may be the small sample size, which is one of the limitations of our research and directly influences the statistical power of the study.

Treatment strategies for MIS-C are based on ongoing surveillance and existing experience with other syndromes that have overlapping features, such as KD, septic shock, and myocardial injury. In most published case series, intravenous immunoglobulin was described as the treatment of choice, followed by corticosteroids in refractory cases [16,21].

Similarly, in our study, all of the MIS-C patients received standardized treatment courses with steroids, IVIG, or IVIG combined with steroids. It is essential that, based on the published literature, ventricular dysfunction improves in the majority of cases in the first week of treatment [8]. All patients (100%) involved in a specific study [22] were treated with IVIG at the time of diagnosis, with a median delay of five +/- two days from the onset of symptoms. Treatment with steroids (methylprednisolone) was added for 30 patients (93.8%).

In our cohort, the majority of patients were treated with immunomodulatory drugs, most commonly systemic glucocorticoids (prednisolone; n = 39, 68.4%), for five weeks; three patients (5.3%) received IVIG with steroids, while two (3.5%) received only IVIG.

Grimaud et al. reported 20 children with shock and acute myocarditis and an LVEF of 35% (IQR, 25-55%); all children received intravenous immunoglobulin (2 g per kilogram) with adjuvant corticosteroids (n = 2), IL-1 receptor antagonist (n = 1), or a monoclonal antibody against the IL-6 receptor (n = 1). All children survived and were afebrile with a full LV function recovery. Overall, according to the literature and our study observations, all the acute cardiovascular manifestations have been successfully resolved after a specific steroid treatment course [23]. In our cohort, it is hard to say what the average timeline of normalization of the clinical findings was because of the lack of timely follow-up sessions from the patients’ side, presenting another limitation of the study.

Our retrospective cohort study has certain limitations. Cases of MIS-C were identified only at M.Iashvili Children’s Central Hospital, and the results are not generalizable beyond the surveillance population. Although we used a database from a single hospital, clinical management was slightly different among patients, including detailed echocardiographic data and laboratory tests performed. Because of the limitations of retrospective database review and clinical testing, we were not able to standardize the laboratory findings of all patients.

Considering the high prevalence of cardiac manifestations in MIS-C patients according to various case series, available literature, and our current study and considering that patients could be asymptomatic, it is our recommendation that MIS-C patients, regardless of their clinical manifestations, be assessed for cardiac abnormalities. This is also in line with the recommendation given by the American Academy of Pediatrics that "any child sick enough to warrant admission for fever, abdominal pain, diarrhea, and/or organ dysfunction in whom MIS-C is suspected should be cared for in a hospital with tertiary pediatric and cardiac intensive care units" [21]. Similar studies like that of Wu and Campbell also recommend that "a thorough cardiac evaluation, including troponin and BNP levels, an electrocardiogram (ECG), and transthoracic echocardiogram should be urgently obtained" as soon as MIS-C is suspected [11].

## Conclusions

To conclude, MIS-C commonly affects the cardiovascular system and leads to variable cardiac manifestations, most of which resolve with specific treatment courses and, fortunately, do not lead to major sequelae. However, little is known about the exact immunopathology of MIS-C, and even less can be elaborated on the long-term cardiac manifestations of the disease. Systematic longer-term follow-up as well as standardized approaches to coronary artery imaging and interpretation are needed to provide clarity on the evolution of medium- and long-term cardiac outcomes in MIS-C.
